# Determinants of neonatal mortality in rural Northern Ethiopia: A population based nested case control study

**DOI:** 10.1371/journal.pone.0172875

**Published:** 2017-04-18

**Authors:** Robel Yirgu, Mitike Molla, Lynn Sibley

**Affiliations:** 1 Department of Reproductive Health and Health Service Management, School of Public Health, College of Health Sciences, Addis Ababa University, Addis Ababa, Ethiopia; 2 Department of Preventive Medicine, School of Public Health, College of Health Sciences, Addis Ababa University, Addis Ababa, Ethiopia; 3 Nell Hodgson Woodruff School of Nursing and Rollins School of Public Health, Emory University, Atlanta, Georgia, United States of America; Centre Hospitalier Universitaire Vaudois, FRANCE

## Abstract

**Introduction:**

In low income and middle income countries, neonatal mortality remains high despite the gradual reduction in under five mortality. Newborn death contributes for about 38% of all under five deaths. This study has identified the magnitude and independent predictors of neonatal mortality in rural Ethiopia.

**Methods:**

This population based nested case control study was conducted in rural West Gojam zone, Northern Ethiopia, among a cohort of pregnant women who gave birth between March 2011 and Feb 2012. The cohort was established by Maternal and Newborn Health in Ethiopia Partnership (MaNHEP) project in 2010 by recruiting mothers in their third trimester, as identified by trained community volunteers. Once identified, women stayed in the cohort throughout their pregnancy period receiving Community Maternal and Newborn Health (CMNH) training by health extension workers and community volunteers till the end of the first 48 hours postpartum. Cases were 75 mothers who lost their newborns to neonatal death and controls were 150 randomly selected mothers with neonates who survived the neonatal period. Data to identify cause of death were collected using the WHO standard verbal autopsy questionnaire after the culturally appropriate 40 days of bereavement period. Binomial logistic regression model was used to identify independent contributors to neonatal mortality.

**Result:**

The neonatal mortality rate was AOR(95%CI) = 18.6 (14.8, 23.2) per 1000 live births. Neonatal mortality declined with an increase in family size, neonates who were born among a family of more than two had lesser odds of death in the neonatal period than those who were born in a family of two AOR (95% CI) = 0.13 (0.02, 0.71). Mothers who gave birth to 2–4 AOR(95%CI) = 0.15 (0.05, 0.48) and 5+ children AOR(95%CI) = 0.08 (0.02, 0.26) had lesser odds of losing their newborns to neonatal mortality. Previous history of losing a newborn to neonatal death also increased the odds of neonatal mortality during the last birth AOR (95%CI) = 0.25 (0.11, 0.53).

**Conclusion:**

The neonatal mortality rate in our study was three times lower than the regional neonatal mortality rate estimate, indicating community based interventions could significantly decrease neonatal mortality. The identified determinants, which are amenable for change, emphasize the need to improve quality of care during pregnancy, labour and delivery to improve pregnancy outcome.

## Introduction

Neonatal mortality, which occurs during the first 28 days after birth contributes to 38% of all under five deaths [1]. Global estimates indicate that annually 4 million neonatal deaths take place among 130 million births [1]. Looking into the global distribution of neonatal mortality, 99% of the deaths take place in low and middle income countries of south-central Asia and sub-Saharan Africa [1]. For the past fifteen years, a global effort was put in place to alleviate poverty and improve health status of children aiming at achieving the Millennium Development Goals (MDGs). This effort managed to decrease under five mortality generally, but significant proportion of this reduction is ascribed to post neonatal mortality [1]. Even though there is a global decrease in neonatal mortality, the rate of decrement is considerably lower than that of the post neonatal under five mortality [2]. As most of the sub-Sahran Africa countries, a decline in neonatal mortality is recorded in Ethiopia, and the nation has achieved MDG-4(reducing under five mortality by two thirds). However, neonatal mortality rate is still unacceptably high at 37 per 1000 live births exceeding the global estimate of 19 per 1000 live births in two folds [2–5]. Furthermore, the annual reduction in neonatal mortality rate since 1995 was only by 1.9%, indicating a similar pattern of lesser neonatal mortality rate reduction [6]. When the national neonatal mortality estimate is disaggregated into regions the Amhara Regional State, where this study was conducted, stands second with 54 per 1000 live births [5]. This estimate is the highest even for high mortality burden sub-Saharan Africa countries [7].

The sustainable development goal (SDG) is aiming for an under five mortality rate of 25 per 1000 live births or less by 2030[8]. To achieve this target, countries with high burden of under-five mortality, such as Ethiopia, must significantly increase their pace. The lessons from the past 15 years emphasizes on the need to concentrate efforts on the neonatal period in order to bring about the aspired reduction in under five mortality [2]. Unfortunately, the existing evidence on neonatal mortality is too limited to design context oriented interventions [1]. Therefore, this study is intended to generate district level estimate and identify determinants of neonatal mortality in rural Ethiopia.

## Methods

### Study design and study area

We conducted a population-based nested case control study among a cohort of pregnant women in three districts of West Gojam zone (North Achefer, South Achefer and Mecha). The zone is located 500 kms away north of the capital city Addis Ababa. Twenty-four kebles (i.e. the smallest administrative unit) were selected from the three districts (7 from North and South Achefer districts each and 10 from Mecha district). The selected three districts were among the highly populous districts of the zone with a total population count of 292,250 in Mecha, 155,863, in South Achefer and 173,211 in North Achefer districts [9]. Each kebele has one health post, providing disease prevention and health promotion services to 2500–5000 population. Each health post is staffed with two health extension workers (HEWs) and reports to the next level called health centers [10].

### Study setting and establishment of the cohort of pregnant women

The cohort was first established in 2010 by Maternal and Newborn Health in Ethiopia Partnership (MaNHEP) project. The project was led by Emory University in collaboration with the Ethiopian Federal Ministry of Health and Addis Ababa University [11]. The intervention implemented Community Maternal and Newborn Health (CMNH) care package through the existing Health Extension Program (HEP) in six woredas (districts) of Amhara and Oromia regions (three from each region) [11,12]. The project was aimed at improving the capability and performance of HEWs to provide targeted maternal and newborn health (MNH) services; increasing demand for targeted MNH services and improve self-care behavior and quality of MNH services in lead woredas. A lead woreda is the one that has the commitment and capacity to continuously improve MNH service delivery to meet the needs of mothers and children [12].

The project used three basic approaches to achieve its objectives: 1) Behavioral change communication on issues of maternal and newborn health, 2) Maternal and newborn health training for health extension workers, women in reproductive age and potential care givers among family members and 3) Continuous collaborative quality improvement interventions [11].

Pregnant women, in their third trimester, were enrolled into the cohort as identified by community volunteers who had CMNH training by MaNHEP. Once in the cohort, mothers received continuous training on care during pregnancy, labour and delivery by the volunteers. This study focused on data collected from mothers in Amhara Region who gave birth between March 2011 and February 2012.

### Study participants

**Fig 1:**
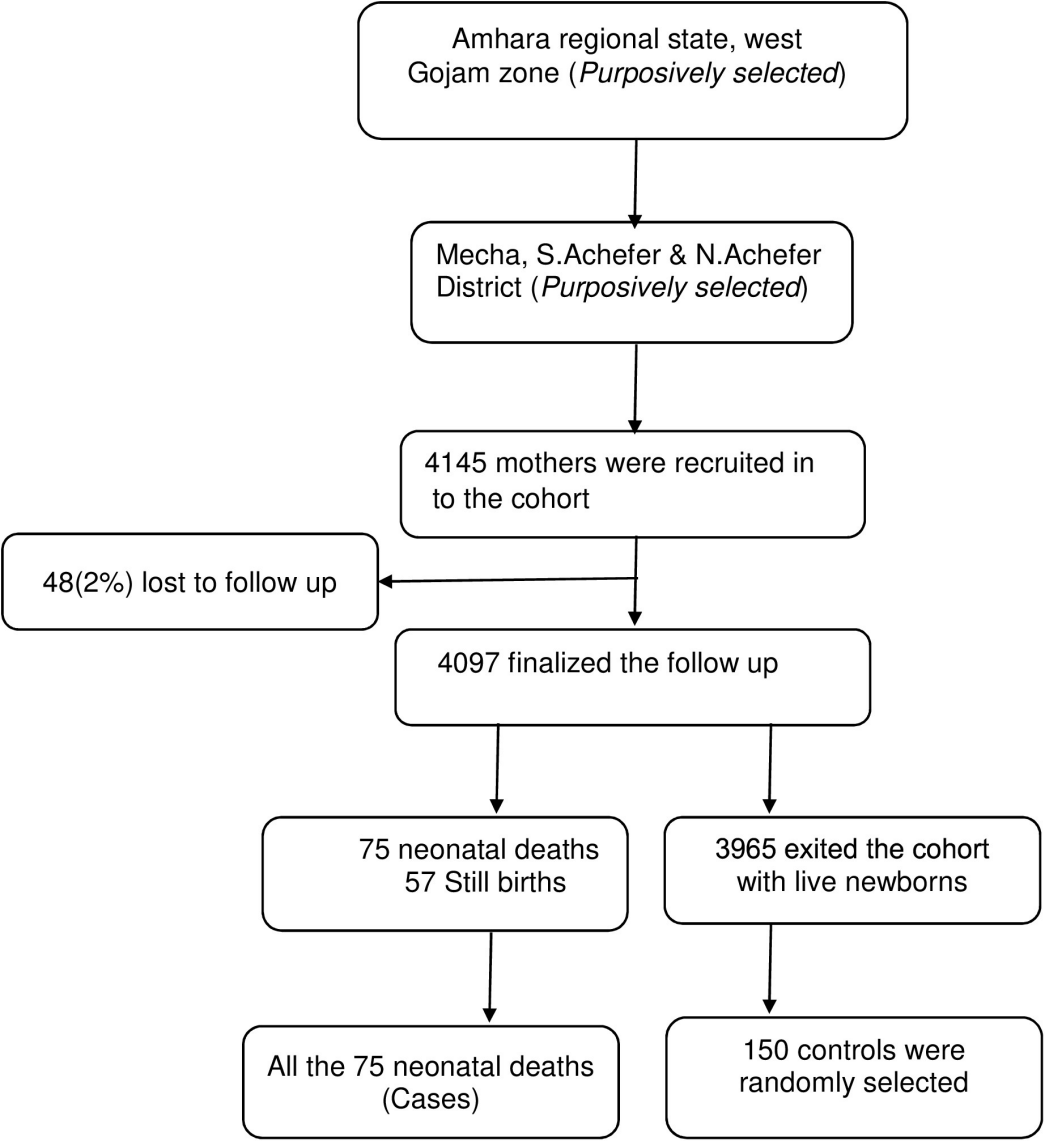
Sample selection procedure.

All pregnant women in their third trimester living in the selected kebeles were recruited into the cohort. The cases were mothers who lost their babies for neonatal death at the end of the follow up period and the controls were mothers with a live neonate at the end of the fourth week after birth. The controls were randomly selected from a sampling frame containing list of mothers, in the respective gotes (i.e. a structure smaller than kebele) of the cases, whose pregnancy outcome was confirmed (Fig 1).

### Variables and data collection

Age of the mother, maternal and paternal education were among the socio-demographic variables involved in the analysis (Table 1). Three high school graduate females trained for five days on the data collection tool and interview techniques collected the data. Mothers who lost their newborns were interviewed earliest at forty days after death of the newborn to minimize recall bias.

**Table 1:** Operational definition and categorization of variables used in the analysis West Gojam zone, Feb 2012.

Variables	Operational definition and categories
Neonatal mortality	Death of a newborn in the first 28 days after birth.
Age of the mother	Maternal age was counted in completed years.
Maternal and paternal education	The level of education parents of the newborn were at during the time of the survey (1 = Illiterate, 2 = Those with primary education or who can read and write)
Maternal occupation	1 = Farmer, are those mothers who regularly take part in activities in the farm; 2 = House wife, when main responsibility in the family is the domestic activity

Family size	Number of family members not including the deceased newborns.
Number of deliveries	Total number of babies the woman gave birth to at or after the 28^th^ gestational week, including the last pregnancy.
History of abortion	The expulsion of product of conceptus tissue before the 28^th^ gestational week.

History of neonatal death	History of the death of ones own newborn in the neonatal period.
Pregnancy related complications	1 = Yes, those who respond yes to any of the danger signs of pregnancy; 2 = No, if the mother doesn’t admit any of the danger signs
Birth spacing	The time gap between the last and the preceding pregnancy.

### Statistical analysis

Data were cleaned and entered using EpiData *version 3*.*1 statistical* software. Further cleaning and analysis was done using SPSS *version 20* statistical software. Frequency and proportion were calculated for all variables which were included in the analysis. Neonatal mortality rate was calculated per 1000 live births. Bivariate analysis was conducted to measure the association between the dependent and individual independent variables. To control the effect of confounding variables multiple binary logistic regression models were used. Crude and adjusted OR with 95% CI (Confidence interval) was used to interpret findings of the bivariate and multivariate analysis respectively.

### Ethical consideration

Ethical clearance was obtained from School of Public Health, College of Health Sciences, Addis Ababa University, Research and Ethical Committee (REC). After the purpose of the study was explained, participants provided a written consent before the interview. Mothers who lost their newborns were interviewed after forty days of culturally acceptable bereavement period.

## Result

### Participants profile and neonatal mortality rate

A total of 4097 mothers were recruited into the cohort for follow-up. Forty-eight (1.2%) of the participants were lost to follow up and the status of their pregnancy outcome could not be ascertained. All mothers (n = 75) who lost their newborn for neonatal death and 150 controls participated in the study giving a response rate of 100%. One hundred seventy-three (76.9%) of the respondents were mothers, 23 (10.2%) were fathers while the rest were grandparents of the newborns. Among mothers who made it to the end of the follow up period, 75 neonatal deaths and 57 still births were registered. The neonatal mortality rate was AOR(95%CI) = 18.6 (14.8, 23.2) per 1000 live births (Table 2).

**Table 2:** Neonatal mortality rate across selected districts of West Gojam zone, Feb 2012.

District	Pregnant mothers	Still birth		Neonatal mortality		Neonatal mortality rate
		no	%	no	%	
Mecha	1818	38	66.7	36	48.0	21.3 (14.4, 27.6)
South Achefer	1217	5	8.8	12	16.0	9.9 (4.3, 15.5)
North Achefer	1062	14	24.6	27	36.0	25.8 (15.6, 34.4)
**Total**	**4097**	**57**	**100%**	**75**	**100%**	**18.6 (14.8, 23.2)**

### Determinants of neonatal mortality

Controlling for potential socio-demographic confounding variables such as; age of the mother, maternal education and maternal occupation and family size were independently associated with neonatal mortality. Newborn babies who were born among a family of more than two had lesser odds of death than those who were born in a family of two, AOR (95% CI) = 0.13(0.02, 0.71) and AOR (95% CI) = 0.04(0.01, 0.27) for those who were born in a family of 3–5 and 6+ respectively (Table 3).

**Table 3:** Socio-economic and demographic factors associated with neonatal mortality, West Gojam zone, Feb 2012 (n = 225).

Variables	Cases(n = 75)		Controls(n = 150)		OR (95% CI)	AOR (95% CI)
	n	%	n	%		
**Age of the mother**						
18–24	13	17.6	10	6.7	1	1
25–29	27	36.7	63	42.0	0.33(0.13, 0.84)	0.51(0.17, 1.47)
30–34	24	32.4	58	38.7	0.32(0.12, 0.83)	0.65(0.21, 2.02)
35–40	10	13.5	19	12.7	0.41(0.13, 1.25)	0.98(0.26, 3.71)
**Maternal education**						
Illiterate	73	97.3	145	97.3	1	1
Primary education and above	2	2.7	4	2.7	0.99(0.18, 5.55)	0.33(0.02, 4.69)
**Paternal education**						
Illiterate	69	92.0	135	90.6	1	1
Primary education and above	6	8.0	14	9.4	0.84(0.31, 2.28)	0.74(0.19, 2.95)
**Maternal occupation**						
Farmer	38	50.7	56	37.3	1	1
House wife	37	49.3	94	62.7	0.58(0.33, 1.02)	0.56(0.31, 1.05)
**Family size**						
2	8	10.7	2	1.3	1	1
3–5	46	61.3	66	44.0	0.17(0.04, 0.86)	0.13(0.02, 0.71)
6+	21	28.0	82	54.7	0.06(0.01, 0.32)	0.04(0.01, 0.27)

^OR^ Odds ratio

^AOR^ Adjusted odds ratio

Among the obstetric related variables number of delivery and previous history of losing a newborn to neonatal death were significantly associated with neonatal mortality. Mothers who gave birth to 2–4 and 5+ children had lesser odds of losing their newborns for neonatal death AOR (95% CI) = 0.15(0.05, 0.48) and AOR (95% CI) 0.08(0.02, 0.26) respectively. Previous history of neonatal death also increased the odds of neonatal mortality during the last birth AOR (95% CI) = 0.25 (0.11, 0.53) (Table 4)

**Table 4:** Present and past obstetric condition related variables and neonatal mortality in West Gojam zone, Ethiopia Feb 2012(n = 225).

Variables	Cases (n = 75)		Controls (n = 150)		OR(95%CI)	AOR(95%CI)
	n	%	n	%		
**Sex of the new born**						
Male	42	56.0	88	58.7	1	1
Female	33	44.0	62	41.3	1.1(0.64,1.95)	1.17(0.61,2.23)
**Number of delivery**						
One	15	20.0	8	5.5	1	1
2–4	33	44.0	55	37.7	0.32(0.12,0.84)	0.15(0.05,0.48)
≥5	27	36.0	83	56.8	0.17(0.06,0.45)	0.08(0.02,0.26)
**History of abortion**						
Yes	12	16.0	13	8.8	1	1
No	63	84.0	135	91.2	0.51(0.22,1.17)	0.53(0.2,1.41)
**History of neonatal death**						
Yes	26	34.7	20	13.3	1	1
No	49	65.3	130	86.7	0.29(0.15,0.57)	0.25(0.11,0.53)
**Birth spacing**						
< 2years	11	14.7	22	14.9	1	1
≥ 2years	64	85.3	126	85.1	1.02(0.46,2.23)	1.71(0.63,4.64)
**Complications**						
Yes	7	9.5	10	6.8	1	1
No	67	90.5	138	93.2	0.69(0.25,1.9)	1.13(0.31,4.19)
**Type of delivery**						
Singleton	69	92.0	143	96.0	1	1
Twin	6	8.0	6	4.0	2.07(0.65,6.66)	1.15(0.26,5.02)
**Place of delivery**						
Home	67	90.5	143	96.6	1	1
Health facility	7	9.5	5	3.4	2.9(0.92,9.76)	2.66(0.72,9.83)

## Discussion

In this study, we found a significantly lower neonatal mortality rate compared to the national and Amahra Regional State estimates. Newborns in a family of two had a higher odd of death in the neonatal period as compared to those who were born in a family of three or more. Previous history of neonatal mortality and number of delivery (parity) were independently associated with neonatal death.

In Ethiopia, the national neonatal mortality rate was 37 per 1000 live births with a gradual decrease from 39 per 1000 in the year 2005[4,5]. The estimated neonatal mortality rate in our study was smaller than both the sub-Saharan Africa regional estimates (29 per 1000 live births) and the Amhara regional state estimates which was 54 per 1000 live births [5,13]. The fact that this study was conducted in an area of community based maternal and child health intervention project might have contributed for the smaller neonatal mortality rate estimates. In the study area, the interventions had improved the knowledge and skill of community health care providers towards CMNH care services [14]. In addition, care seeking for targeted maternal and newborn health care services from trained providers had significantly improved by the end of the intervention period [11]. But difference in mortality rate was observed among the districts. The high neonatal mortality rate in North Achefer district can be attributed to the arduous topography and remoteness of the kebeles in the district, discouraging facility delivery specially during night time. Kebeles in South Achefer district have a relatively better access to local health centers, in addition this district is even closer to the nearest referral hospital than North Achefer district. In this study, newborn babies who were born in a family of two had higher odds of death in the first four weeks of birth. Family size is among the known determinants of neonatal death, large family size and growing among extended family members increases the risk of dying in the neonatal period [15]. On the contrary, our study indicated that smaller family size increased the likelihood of death. We assume participants who claimed to have a family size of only two were newlyweds who gave birth to their first child. This association was again reflected in the second model (number of deliveries) where first born children had higher odds of death than their second or third born counterparts. In this cultural community, it is customary to give birth soon after getting married [16]. Therefore, most of the mothers who gave birth to only one child were presumed to be young mothers who suffered one of the risks of primiparity, which is immediate newborn death [17].

This argument also justifies the increased odds of death among neonates born to nulliparas in the second model. In addition, women who get pregnant for the first time often give birth at their mothers’ homestead, this might have decreased their chance of getting help from the intervention kebeles, which could have increased the risk of neonatal death.

The association between previous history of neonatal death and mortality of the index case is also seen in other studies [17–20]. The prevailing newborn mishandling compounded of traditional practices, had claimed the lives of newborns in the area [16]. These practices possibly contributed for the repeated newborn deaths in a family. Pregnancy related complications including adverse pregnancy outcomes during previous pregnancies signify the need for a close follow up during pregnancy and child birth [19]. The health extension program (HEP) has improved access to primary health care in rural Ethiopia [19]. However, this program is mainly established for promotive and preventive health care services but not designed to provide delivery care. Therefore, high risk pregnant women should be referred early to the higher-level health facility to obtain a better care during pregnancy and childbirth.

Some of the strengths of this study include employing a nested case control study, a design which is robust to identify determinants of neonatal mortality. We have followed a fairly large cohort of pregnant mothers, which added to the power of the study in identifying determinant factors and give a more precise estimate of mortality rates. On the other hand, due to the nature of the design, our study might not have adequately addressed the contribution of socio-cultural factors which affect neonatal mortality. Further qualitative study could have given a better understanding of the sociocultural factors contributing to neonatal mortality.

## Conclusion

The neonatal mortality rate estimate in our study is high, but it is considerably lower than the national and regional estimates. This decline in mortality suggested that simple community based interventions could significantly reduce newborn death. Factors such as number of deliveries and previous obstetric complications have contributed for most of the identified deaths. Improving the quality of a targeted care for mothers during pregnancy and childbirth can reduce deaths associated with preventable risk factors.

## Supporting information

S1 Supporting Information Interview questionnaire. (DOC)
