# Supplementary material for: Determinants of neonatal mortality in rural Northern Ethiopia: A population based nested case control study
Source: PLoS One. 2017 Apr 18;12(4):e0172875. doi: 10.1371/journal.pone.0172875 (PMC5395163; doi:10.1371/journal.pone.0172875)
Supplement: S1 Supporting Information — (DOC) [file pone.0172875.s001.doc]

***Instructions to interviewer: Introduce yourself and explain the purpose of your visit. Ask to speak to the mother or to another adult caretaker who was present during the illness that led to death. If this is not possible, arrange a time to revisit the household when the mother or caretaker will be home.***

**INTERNATIONAL STANDARD VERBAL AUTOPSY QUESTIONNAIRE**

DEATH OF A CHILD UNDER 4 WEEKS

| **SECTION 1 INTERVIEWER VISITS Interviewer Put 1 for cases and 2 for referents in the Box** | | | | | | | |
| --- | --- | --- | --- | --- | --- | --- | --- |
| INTERVIEWER VISITS | **1** | **2** | | **3** | | **FINAL VISIT** | |
| DATE  INTERVIEWER NAME  RESULT CODE | ……………………  ……………………… | ……………………..  ……………………… | | ……………………..  ……………………… | | DAY……………………  MONTH  YEAR  INT. NUMBER  RESULT……….…… | |
| NEXT VISIT:  DATE  TIME | ……………………  ………………… | ……………………..  ……………………… | | ……………………..  ……………………… | | TOTAL NUMBER OF VISITS  ………………..………… | |
| **RESULT CODES**  1=COMPLETED 3=POSTPONED 5=PARTLY COMPLETED  2=NOT AT HOME 4=RESPONDENT REFUSED 6 =NO APPROPRIATE RESPONDENT FOUND  7 = OTHER (SPECIFY)_______________________________ | | | | | | | |
| SUPERVISOR  Name ______________  Date _______________ | | | FIELD EDITOR  Name ______________  Date _______________ | | OFFICE  EDITOR | | KEYED BY |
| PLACE NAME/ LOCATION_________________  ADDRESS LOCATION TO THE HOUSEHOLD_____________________________________________________________  _________________________________________________________________________________________________ | | | | | | | |
| INTERVIEWERS NAME SIGNATURE AND DATE.....................................................  SUPERVISOR SIGNATURE AND DATE……………………………….………  DATA ENTRY SIGNATURE AND DATE……………………………………….. | | | | | ………………………..  ………………………… | | |
| Hello my name is _________________________________and am working with MaNHEP  We are collecting information on the causes of death in the community. We would very much appreciate your participation in this effort. We want to ask you about the circumstances leading to the death of the deceased. Whatever information you provide will be kept strictly confidential. No information identifying you or the deceased will ever be realized to anyone outside of this information –collection activity.  Participation in this survey is voluntary and you can choose not to answer any individual question or all of the questions. You may also stop the interview completely at any time without any consequences at all. However, we hope that you will participate in this survey since the results will help the government improve service for people. If you have any questions about the study feel comfortable to raise it so that I will try to give you the answers ,even if I do not have the answers I will give you the contact address of the research team for you to be enlightened with the details of the study. Your participation in this study doesn’t involve any payment on either sides.  At this time, do you want to ask me anything about the purpose or content of this interview?  May I began the interview now?  Signature of interviewer: ___________________________date ___________________________  Respondent Agree to be interviewed……………………….1  Respondent do not agree to be interviewed-----------------2 END THE INTERVIEW | | | | | | | |

## District____________________________

## Kebele___________________________

## Gote_____________________________

##

## **SECTION 2. BASIC INFORMATION ABOUT THE RESPONDENT**

| 201 Record the time at start of interview | | Hour  Minutes |  |  |
| --- | --- | --- | --- | --- |
|  |  |
| 202 What is the name of the main respondent: |  | | | |

203 What is the relationship of the main respondent with (name of the child) child? (Circle relevant number)

| 1. Father | 2. Mother | | 3. Sibling | | 4. Grand parent | | 5. Other relative | | | | | 6.No relation | | |
| --- | --- | --- | --- | --- | --- | --- | --- | --- | --- | --- | --- | --- | --- | --- |
| *6. Other (specify):* | | | | | | | | | | | | | | |
| 204. Can the respondent read and write? | | 1. Yes | | 2. No | | | | | | 206 | | | | |
| 205. How many years of school did the main respondent  complete? | | | | 1. Grade-1 6.Grade-6 11.Grade-11 2. Grade-2 7.Grade-7 12.Grade-12 3. Grade-3 8.Grade-8 13.10^+^Diploma 4. Grade-4 9.Grade-9 14.12^+^Diploma 5. Grade-5 10.Grade-10 15.University | | | | | | | | | | |
| 206. Were other people present at the interview? …………………… | | | | | | 1.Yes | | | 2. No | | | |  | |
| If the answer is yes who what is their relation with the newborn? | | | | | | Present during the time of | | | | | | | | |
|  |  |  |  |  |  | Interview | | | | | Illness(if any) | | | |
|  |  |  |  |  |  | Yes | | No | | | Yes | | | No |
| 1. Father | | | | | | 1 | | 2 | | | 1 | | | 2 |
| 1. Mother | | | | | | 1 | | 2 | | | 1 | | | 2 |
| 1. Grand mother | | | | | | 1 | | 2 | | | 1 | | | 2 |
| 1. Grand father | | | | | | 1 | | 2 | | | 1 | | | 2 |
| 1. siblings | | | | | | 1 | | 2 | | | 1 | | | 2 |
| 1. Aunt/uncle | | | | | | 1 | | 2 | | | 1 | | | 2 |
| 1. Relatives | | | | | | 1 | | 2 | | | 1 | | | 2 |
| 1. Neighbours | | | | | | 1 | | 2 | | | 1 | | | 2 |
| 1. Others______________________________ | | | | | |  | |  | | |  | | |  |
| 207. Name of the deceased or the control ____________________________________________________________ | | | | | | | | | | | | | | |
| 208. Age of the deceased or the control (in days) | | | | | |  | | | | | | | | |
| 209. Sex of the child | | | | | | 1. Male 2. Female | | | | | | | | |

SECTION 3 - LIVELIHOODS AND HEALTH SERVICES

| 301. What is the main source income for the family?  ***PROBE TWO TIMES ‘ANYTHING ELSE?’ CIRCLE ALL THAT APPLY HERE IT IS ALLOWED TO ENCIRCLE MORE THAN ONE OPTION*** | | | | | | 1 =Farming  2 = Domestic worker/cleaner  3 = Construction work  4 = Petty trader  5 = Dressmaker/hairdresser  6 = Waitress / barmaid  7 = Financial support from families or children abroad  8.= Financial support from children or other families in the country  9 = Making tela, areke, other alcoholic drink  10 =Plumber, elect, carpenter,mechanic, etc.  11 = Professional (health care, teacher, manager, etc.)  12= Pension  13 = Any other source of income? (Specify) ……………………….. | | | | | | |
| --- | --- | --- | --- | --- | --- | --- | --- | --- | --- | --- | --- | --- |
| .  302. Do you rent, own, or are you provided with the place/house  that you live? | | | | | | 1 = Owned  2 = Provided by employer  3 = Provided by relatives  4 = Rented from employer  5 = Rented from kebele  6 = Rented from private owners  7 = Rented from other (Specify) ………………………… | | | | | | |
| 303. How many rooms does the household have? | | | | | | Number of rooms | | | | | | |
| 304. Is there a separate place for cattle and people? | | | | | | 1. 1=Yes   2=No | | | | | | |
| 305.Is there a separate kitchen from the main household? | | | | | | 1. 1=Yes 2. 2=No | | | | | | |
| 306. Number of windows in the household? | | | | | |  | | | | | | |
| .  307. What is the main material of the roof? | | | | | | 1=Corrugated iron sheet  2 =Thatch or grass  3 = Other (specify) ……………………… | | | | | | |
| .  308. What is the main material of the floor? | | | | | | 1.Earthen floor  2.Cement  3. Straw  4.Plastic tiles  5.Plastic sheet  6.wood | | | | | | |
| 309. How often do you cook in the house? | | | | | | 1. All the time  2. Some times | | | | | | |
| .  310. What is the main type of cooking fuel? | | | | | | 1 = Mainly collected firewood  2 = Mainly purchased firewood  3 = Charcoal  4 = Kerosene  5 = Butane gas  6 = Electricity  7 = Leaves/dung cakes  8 = Other (specify) ………………………… | | | | | | |
| 311.Is there a toilet facility? | | | | | | 1. 1=Yes   2=No | | | | 314 | | |
| .  312. What type of toilet facility does the household have? | | | | | | 1 = Flush toilet, private  2 = Flush toilet, shared with another household  3 = Pit latrine, private  4 = Pit latrine, shared  5 = Container (from household items)  6 = Field/forest  7 = Other (specify) …………………………………… | | | | | | |
| 313. Does the family use the toilet regularly? | | | | | | 1. Yes always  2. Some times  3. Never | | | | | | |
| .  314. What is the main source of drinking water? | | | | | | 1 = Tap water  2 = Protected well/spring  3 = Unprotected well/spring  4= Rain water  5 = River, lake or pond | | | | | | |
| **I want to talk about the items that a household or individual might own or possess. I will ask you about what your current household own.** | | | | | | | | | | | | |
| 315. Does any member of the household own or rent any land that can be used for agriculture | | | | 1 = Yes  2= No | | | | 317 | | | | |
| 316. How many (Local Units) of agricultural land do members of household own? | | | | In Local Units_______________ | | | | | | | | |
| 317. Does this household own any livestock, heard, or farm animals? | | | | 1 = Yes  2= No | | | | 319 | | | | |
| 318. How many of the following animals does this household own? | | | | Bulls................................................................................................  Oxen.............................................................................................  Cows............................................................................................  Donkeys.......................................................................................  Horses........................................................................................  Mule.........................................................................................  Goat................................................................................  Sheeps......................................................................................  Cock/hens...................................................................................... | | | | | | | | |
| ***319. READ EACH ITEM. IF ITEM IS OWNED BY HH CIRCLE ‘1’. IF ITEM IS NOT OWNED BY THE HH***  ***CIRCLE ‘2’.*** | | | | Item | | | | | Yes | | | No |
|  |  |  |  | **A=**Electricity | | | | | 1 | | | 2 |
|  |  |  |  | B = Radio | | | | | 1 | | | 2 |
|  |  |  |  | C= Television | | | | | 1 | | | 2 |
|  |  |  |  | D=Telephone | | | | | 1 | | | 2 |
|  |  |  |  | E.=Mobile phone | | | | | 1 | | | 2 |
|  |  |  |  | F =Electric Mitad | | | | | 1 | | | 2 |
|  |  |  |  | G = Kerosene Lamp | | | | | 1 | | | 2 |
|  |  |  |  | H = Bed. | | | | | 1 | | | 2 |
|  |  |  |  | I = Plough | | | | | 1 | | | 2 |
|  |  |  |  | J = Tractor | | | | | 1 | | | 2 |
|  |  |  |  | K = Motor cycle | | | | | 1 | | | 2 |
|  |  |  |  | L= Cart | | | | | 1 | | | 2 |
|  |  |  |  | M= Cash Crops, such as coffee, sesame, etc.. | | | | | 1 | | | 2 |
|  |  |  |  | N= Tables | | | | | 1 | | | 2 |
|  |  |  |  | O.= Refrigerator | | | | | 1 | | | 2 |
|  |  |  |  | P= Stove/electric /gas | | | | | 1 | | | 2 |
|  |  |  |  | Q= Car/Van | | | | | 1 | | | 2 |
|  |  |  |  | R= Chairs | | | | | 1 | | | 2 |
| 320. Do you have any personal cash savings you keep for future plans or in case of emergencies? | | | | | | 1 = Yes  0 = No | | | | | | 323 |
| .  321. Where do you currently keep your savings?  ***PROBE TWO TIMES ‘ANYTHING ELSE?’ CIRCLE ALL THAT APPLY*** | | | | | | 1 = Bank  2 = Micro finance organization  3 = Ekub  4 = Mobile bank  5 = At home  6 = With a friend  7 = Other (specify) ……………………………………… | | | | | | |
| 322. Approximately how much personal savings do you currently have? | | | | | | |____|____|____|____| BIRR 98=Non-response | | | | | | |
| 323. If the family faced a financial problem for health care, is there any source that can lend the HH money ? | | | | | | 1. Certainly  2. I think so  3. I do not think so  4. Never | | | | | | |
| 324. What is the main income source of the family | | | | | | ------------------------------- | | | | | | |
| 325. Type of health facility in the vicinity | | | | | | 1. Hospital 2. Health centre 3. Private clinic 4. Health post | | | | | | |
| 326. What is the average distance to a nearest HF | | | | | | ___________________ walking distance (in days)  ___________________ walking distance (in hours)  ___________________ in Km | | | | | | |
| 327. What is the average distance to the nearest hospital | | | | | | ___________________ walking distance (in days)  ___________________ walking distance (in hours)  ___________________ in Km | | | | | | |
| 328. Where do you usually seek health care service if someone in the house hold gets sick? | | | | | | | | | | | | |
|  | | 1. Traditional healer | 2. Religious leader | | 3. Public Hospital | | 4. Health centre | | | | 5.Health post | |
|  | | | | | 6. Private clinic | | 7. Pharmacy, drug seller | | | | 8. Holy water | |
|  | Other ( specify) | | | | | | | | | | | |

**SECTION 4** MOTHER'S HEALTH AND CONTEXTUAL FACTORS

| 401 What was the age of the mother at the time the baby was born or died? | | YEARS ………………………….  98.DON'T KNOW | | |  |
| --- | --- | --- | --- | --- | --- |
| 402. What was the marital status of the mother when the baby was born or  died? | | 1.Married  2.Single  3.Divorced  4.Separated  5.Widowed | | |  |
| 403 What is the educational status of the mother? | 1. Grade-1 6.Grade-6 11.Grade-11 2. Grade-2 7.Grade-7 12.Grade-12 3. Grade-3 8.Grade-8 13.10^+^Diploma 4. Grade-4 9.Grade-9 14.12^+^Diploma   5. Grade-5 10.Grade-10 15.University | | | |  |
| 404 What is the educational status of the father? | 1. Grade-1 6.Grade-6 11.Grade-11 2. Grade-2 7.Grade-7 12.Grade-12 3. Grade-3 8.Grade-8 13.10^+^Diploma 4. Grade-4 9.Grade-9 14.12^+^Diploma 5. Grade-5 10.Grade-10 15.University | | | |  |
| 405. Mother’s occupation? | 1.House wife  2.Factory worker  3.Civil servant | | 4.Merchant  5.Farmer  6. Daily labourer  7.Others specify | |  |
| 406. What is your family size? | Numbers .............................. | | | |  |
| 407. Did the mother receive antenatal care? | 1.Yes  2.No  98.Don't know | | | 412 |  |
| 408. When did you start attending ANC? | 1.First three months  2.Second three months  3.Third three months | | | |  |
| 409. Where did you attend ANC service? | 1.Hospital  2.Health centre  3.Health post  4.Private clinic | | | |  |
| 410. Was there any abnormality identified during ANC? | 1.yes  2.No | | | **412** |  |
| 411. Did you get any advice or help for the abnormality? | 1.Yes  2.No  98.Don't know | | | |  |
| 412. Did the mother receive tetanus toxoid (TT) vaccine? | 1.Yes  2.No  98.Don't know | | | |  |
| 413. How many doses? | 1.Number of doses ………….  98.Don't know | | | |  |
| 414. How is the mother’s health now? | 1.Healthy  2.Ill  3.Not alive  98.Don't know | | | |  |
| 415. Have you ever had an abortion? | 1.Yes  2.No  98.Don't know | | | | 417 |
| 416. How many abortions have you had? | Numbers ……………………… | | | |  |
| 417. Have you had a previous neonatal mortality? | 1.Yes  2.No  98.Don’t know | | | |  |
| 418. How many neonatal deaths did you encounter? | Numbers ………………………  98.Don’t know | | | |  |
| 419. Was the child birth preceding your last delivery at  the expected time? | 1.Term  2.Pre term  3.Post term  98.Don’t know | | | |  |

# **SECTION 5:** PREGNANCY HISTORY

| 501. How many births, including stillbirths, did the mother  has before this baby? | | Number of births/stillbirths..................  98.Do not know | | | | | |  |
| --- | --- | --- | --- | --- | --- | --- | --- | --- |
| 502. How many months was the pregnancy when the  (control baby) or the deceased was born? | | Gestational age.......................  98.Do not know | | | | | |  |
| 503. Did the pregnancy end earlier than expected? | | 1.Yes  2.No  98.Don't know | | | | | |  |
| 504. During the pregnancy did the mother suffer from any  of the following known illnesses:  1 High blood pressure?  2 Heart diseases?  3 Diabetes?  4 Epilepsy/convulsion?  5 Did she suffer from any other medically  diagnosed illness? | | |  | | | | | --- | --- | --- | --- | |  | **Yes** | **No** | **DK** | | High blood pressure? | 1 | 2 | 98 | | Heart diseases? | 1 | 2 | 98 | | Diabetes? | 1 | 2 | 98 | | Epilepsy/convulsion? | 1 | 2 | 98 | | Did she suffer from any other medically diagnosed illness? | | | | | | | | | |  |
| 505 .During the last 3 months of pregnancy did the mother suffer from any of the following illnesses: |  | | | **Yes** | | **No** | **DK** |  |
|  | 1. Vaginal bleeding?  2. Smelly vaginal discharges?  3.Puffy face?  4. Headache?  5. Blurred vision?  6. Convulsion?  7. Febrile illness?  8. Severe abdominal pain that was not labor pain?  9 .Pallor and shortness of breath (both present)?  10.Did she suffer from any other illness? | | | 1  1  1  1  1  1  1  1  1 | | 2  2  2  2  2  2  2  2  2 | 98  98  98  98  98  98  98  98  98 |  |
|  |  |  |  | Other illnesses specify ____________________ | | | |  |
| 506.Was the child a single or multiple birth? | | | | 1.singleton  2.twin  3.triplet or more  98.don't know | | | |  |
| 507. What was the birth order of the child that was born alive or died? | | | | 1.first  2.second  3.third  4.fourth  5.Fifth or higher  98.Do not know | | | |  |
| 508. How long have you waited to give birth to your last baby after giving birth to the presiding  one? | | | | 1.One year  2.One and half year  3.Two years  4.More than two years  98.dont know | | | |  |
| 509.Have you obtained post natal care in the seven post partum days that followed your last  child birth? | | | | 1.Yes  2.No  98.Don’t know | | | |  |
| **SECTION 6 : DELIVERY HISTORY** | | | | | | | | |
| 601. When did the water break? | | | 1.Before labor started  2.During labor  98.Don't know | | | | |  |
| 602. How many hours after the water broke was the baby born? | | | 1.Less than 24 hours  2.24 hours or more  98.Don't know | | | | |  |
| **Question 603 – 607 only for cases** | | | | | | | | |
| 603. Was the water foul smelling? | | | 1.Yes  2.No  98.Don't know | | | | |  |
| 604. Did the baby stop moving in the womb? | | | 1.Yes  2.no  98.don't know | | | | | 606  606 |
| 605. When did the baby stop moving in the womb? | | | 1.Before labor started  2.During labor  98.Don’t know | | | | |  |
| 606. Were fetal heart sounds present? | | | 1.Yes  2.No  98.Don't know | | | | |  |
| **608- 614 Both cases and controls** | | | | | | | | |
| 607. Was there excess bleeding on the day labor started? | | | 1.Yes  2.No  98.Don't know | | | | |  |
| 608. Did the mother have a fever on the day labor started? | | | 1.Yes  2.No  98.Don't know | | | | |  |
| 609. How long did the labor pains last? | | | 1. Less than 12 hours  2. 12-23 hours  3. 24 hours or more  98. Don’t know | | | | |  |
| 610. Was it a normal vaginal delivery? | | | 1. Yes  2. No  98. Don't know | | | | |  |
| 612. What type of delivery was it? | | | 1. Forceps/vacuum  2. Caesarean section  98. Don't know  Other ……………………………………  (specify) | | | | |  |
| 613. Which part of the baby came first? | | | 1.Head  2.Bottom  3.Feet  4.Arm/hand  98. Don't know  Other ……………………………………  (specify) | | | | |  |
| 614. Did the umbilical cord come out before the baby was born during  labour? | | | 1. Yes  2. No  98. Don't know | | | | |  |
| 615. Where did the mother gave birth to the baby? | | | 1.In the house  2.Health center  3.Hospital | | 4.Health post  5.On the way to a health facility  6.Others_____________________________ | | |  |

***Interviewer section 7 to section 12 is only for Cases ( new born who have died and stillbirths only***

| ***SECTION 7 INFORMATION ON THE DECEASED AND* DATE/PLACE OF DEATH** | | | | | | | | | | | | | | | | | | | | | |
| --- | --- | --- | --- | --- | --- | --- | --- | --- | --- | --- | --- | --- | --- | --- | --- | --- | --- | --- | --- | --- | --- |
| 701 what was the name of the deceased ?  **(*interviewer, use NA for still births)*** | | |  | | | | | | | | | | | | | | | | | |  |
| 702. Sex of the deceased? | | | | 1. Male | | | | | | | 2. Female | | | | | | | | |  | |
| 703. when was the deceased born ?  DD/MM/YY……………………………………………………….  Record 98 if do not know day or month  Record 9998 if do not know year | | | | |  | |  | |  | | | |  | |  | |  | |  | | |
| 704. How old was the deceased when she/he died | | | | | | | | | | | |  | | | |  | | | |  | |
| 705. When did s/he die ……………………………………………………….  Record 98 if do not know day or month  Record 9998 if do not know year | | | | | |  | |  | |  | | | |  | |  | |  | |  | |
| 706. Where did (name of child) die?. | 1. Hospital | 2. Health centre | | | | 3.Health post | | | | | | | | | | | | | | | |
|  | 4. On route to hospital or health facility | | | | | 5. Home | | | | | | | | | | | | | | | |
|  | 6. Other (specify): | | | | |  | | | | | | | | | | | | | | | |

707. For deaths at hospital or health facility, record facility name and address: _______________________________________________________________________________________________________________

**SECTION 8: OPEN HISTORY QUESTION**

801. Could you tell me about his/her illness that led to her/ his death?

Prompt: Was there anything else?

***Instructions to interviewer - Allow the respondent to tell you about the illness in his or her own words. Do not prompt except for asking whether there was anything else after the respondent finishes. Keep prompting until the respondent says there was nothing else. While recording, underline any unfamiliar terms.***

____________________________________________________________________________________________________________________________________________________________________________________________________________________________________________________________________________________________________________________________________________________________________________________________________________________________________________________________________________________________________________________________________________________________________________________________________________________________________________________________________________________________________________________________________________________________________________________________________________________________________________________________________________________________________________________________________________________________________________________________________________________________________________________________________________________________________________________________________________________________________________________________________________________________________________________________________________________________________________________________________________________________________________________________________________________________________________________________________________________________________________________________________________________________________________________________________________________________________________________________________________________________________________________________________________________________________________________________________________________________________________________________________________________________________________________________________________________________________________________________________________________________________________________________________________________________________________________________________________________________________________________________________________________________________________________________________________________________________________________________________________________________________________________________________________________________________________________________________________________________________________________________________________________________________________________________________________________________________________________________________________________________________________________________________________________________________________________________________________________________________________________________________________________________________________________________________________________________________________________________________________________________________________________________________________________________________________________________________________________________________________________________________________________________________________________________________________________________________________________________________________________________________________________________________________________________________________________________________________________________________________________________________________________________________________________________________________________________________________________________________________________________________________________________________________________________________________________________________________________________________________________________________________________________________________________________________________________________________________________________________________________________________________________________________________________________________________________________________________________________________________________________________________________________________________________________________________________________________________________________________________________________________________________________________________________________________________________________________________________________________________________________________________________________________________________________________________________________________________________________________________________

802 Cause of death 1 according to respondent __________________________________________________________________________________________________________________________________________________________________________________________

803 Cause of death 2 according to respondent __________________________________________________________________________________________________________________________________________________________________________________________

***Take a moment to tick all items mentioned spontaneously in the open history questionnaire. Use this to guide you through the rest of the questionnaire****.*

| **804 Symptom** | **Illness day that symptom began** | **Duration of symptom (days)** | **Severity:**  **(Mild-moderate/ Severe)** |
| --- | --- | --- | --- |
| 804.1 |  |  |  |
| 804.2 |  |  |  |
| 804.3 |  |  |  |
| 804.4 |  |  |  |
| 804.5 |  |  |  |
| 804.6 |  |  |  |
| 804.7 |  |  |  |

Note: When possible, use local term for the symptom.

| 805 What was the length of time the child was ill before he/she died? ……… | | | | |
| --- | --- | --- | --- | --- |
| 806 Was care sought outside the home while he/she had this illness? | 1.Yes | 2. No | 3. Don't know | 808 |

*807 (If yes ask:) Where or from whom did you seek care when the child was seek ?* (Record all responses)

| 1. Traditional healer | 2. Religious leader | 3. Government hospital | 4. Government health centre or clinic | | | 5.Health extension worker | | | |
| --- | --- | --- | --- | --- | --- | --- | --- | --- | --- |
| 6. Private clinic | 7. Pharmacy, drug seller | | | | 8. Relative, friend (outside household) | | | | |
| 9. Holy water | | | | Other ( specify) | | | | | |
| 808. What was the length of time of the illness immediately preceding the child death (days) | | | | | | |  | 999.NK | ILLD |

***After respondent finishes prompt:* Did you seek care anywhere else? *Keep using this prompt until respondent replies that they did not seek care from anyone else.***

**SECTION 9 HISTORY OF INJURY/ACCIDENTS**

| 901. Did the baby die from an injury or accident? | | 1.Yes  2.No  98.Don't know | | | 1001 |
| --- | --- | --- | --- | --- | --- |
| 902. What kind of accident did the baby suffer? | | 1.Road traffic accident  2.Fall  3.Drowning  4.Poisoning Burn  5.Violence/assault  6.Bite or sting | | |  |
| 903. Was the injury/ accident inflicted by someone else? | | 1.Yes  2.No  98.Don't know | | | 905 |
| 904.What kind of injury was it? | | 1.Birth trauma  2.Cold injury  3.Suffocation  4.Circumcision | 5.Uvelectomy  6.Tonsilectomy  7.Milk tooth extraction  8.Others_____________ | |  |
| 905.Did the baby suffer from any insect/animal bite that lead to his  death? | | 1.Yes  2.No  98.Don't know | | | 907 |
| 906. What type of animal /insect | | 1.Dog  2.Snake  3.Insect  4.Other  98.Do not know | | |  |
| 907. Did the baby die at the site where the accident occurred? | | 1.Yes  2.No  98.Don't know | | |  |
| 908. For how long after the accident or injury did the baby survive? | | Days  Hrs  Minutes | | |  |
| 909. Did the baby receive medical care before death? | | 1.Yes  2. No  98. Don't know | | |  |
| 910. Did the baby have an ongoing chronic illness or was sick in the month before the accident or injury? | 1.Yes  2.No  98.Don't know | | | 1001 | |
| 911. What was the illness? | _______________________________ | | |  | |

**SECTION 10 CONDITION OF THE BABY SOON AFTER BIRTH**

| 1001. At birth what was the size of the baby? | 1.Smaller than normal  2.Normal  3.Larger than normal  98.Don't know | |  |
| --- | --- | --- | --- |
| 1002. Was the baby premature? | 1.Yes  2.No  98.Don't know | | 1004 |
| 1003. How many months or weeks long was the pregnancy? | Months ……………………………..  Weeks ……………………………...  98.Don't know | |  |
| 1004. What was the birth weight of the baby?  KILOGRAMS | Kilograms ………………………………………  98.Don't know | |  |
| 1005. Was anything applied to the umbilical cord stump after birth? | 1.Yes  2.No  98.Don't know | |  |
| 1006. What was it?  (SPECIFY) | __________________________________________________________________________________ | |  |
| 1007. Were there any signs of injury or broken bones? | 1.Yes  2.No  98.Don't know | | 1009  1009 |
| 1008. Where were the marks/ signs of injury the body? | ____________________________________________________________________________________________________________________________________________ | |  |
| 1009. Was there any sign of paralysis? | 1.Yes  2.No  98.Don't know | |  |
| 1010 Did the baby have any malformations at birth? | 1.Yes  2.No  98.Don't know | | 1012  1012 |
| 1011. What kind of malformation did the baby have? | 1.Swelling/defect on the back  2.Very large head  3.Very small head  4.Defect of lip and/or palate  5.Other malformation  (specify)  98.Don't know | |  |
| 1012. What was the color of the baby at birth? | 1.Normal  2.Pale  3.Blue/black  98.Don't know | |  |
| 1013. Was the baby able to breathe even a little after birth? | 1.Yes  2.No  98.Don't know | |  |
| 1014. Was the baby given assistance to breathe? | 1.Yes  2.No  98.Don't know | |  |
| 1015. Was the baby able to cry even a little at birth? | 1.Yes  2.No  98.Don't know | |  |
| 1016. Did the baby ever move, even a little? | 1.Yes  2.No  98.Don't know | |  |
| 1017. Check codes 1013, 1015 and 1016  All codes ‘NO:  Baby did not breathe  Baby didn’t cry  Baby did not move’ | | | 1101 |
| 1017. If the baby was not breathing, crying or moving. Was it born dead? | | 1.Yes  2.No  98.Don't know | 1101  1101 |
| 1018. Was the baby macerated, that is showing the signs of decay? | | 1.Yes  2.No  98.Don't know | 1101  1101 |

**SECTION 1100 NEONATAL ILLNESS HISTORY**

| 1101. Was the baby ever able to suckle or bottle-feed? | 1.Yes  2.No  98.Don't know |  |
| --- | --- | --- |
| 1102How soon after birth did the baby suckle or bottle-feed? | Hours  Days  Do not know |  |
| 1103. Did the baby stop suckling or bottle-feeding? | 1.Yes  2.No  98.Don't know | 1105  1105 |
| 1104. How many days after birth did the baby stop suckling  or bottle-feeding? | Days……………………………  98.Don't know |  |
| 1105. Was the breastfeeding exclusive | 1.Yes  2.No  98.Don't know |  |
| 1106. Did the baby have spasms or convulsions? | 1.Yes  2.No  98.Don't know | 1108  1108 |
| 1107. How soon after birth did the convulsions start? | Days……………………………  98.Don't know |  |
| 1108. Did the baby become stiff and arched backwards? | 1.Yes  2.No  98.Don't know | 1111  1111 |
| 1109.Did the baby have a bulging of the fontanelle? | 1.Yes  2.No  98.Don't know | 1111  1111 |
| 1110. How many days after birth did the baby have the bulging? | Days……………………………  98.Don't know |  |
| 1111. Did the baby become unresponsive or unconscious? | 1.Yes  2.No  98.Don't know | 1113  1113 |
| 1112. How many days after birth did the baby become  unresponsive or unconscious?' | Days……………………………  98.Don't know |  |
| 1113. Did the baby have a fever? | 1.Yes  2.No  98.Don't know | 1115  1115 |
| 1114. How many days after birth did the baby have a fever? | Days……………………………  98.Don't know |  |
| 1115. Did the baby become cold to the touch? | 1.Yes  2.No  98.Don't know | 1117  1117 |
| 1116. How many days after birth did the baby become cold to the touch? | Days……………………………  98.Don't know |  |
| 1117. Did the baby have a cough? | 1.Yes  2.No  98.Don't know | 1119  1119 |
| 1118. How many days after birth did the baby start to cough? | Days……………………………  98.Don't know |  |
| 1119. Did the baby have fast breathing? | 1.Yes  2.No  3.Don't know | 1121  1121 |
| 1120. How many days after birth did the baby start breathing fast? | Days……………………………  98.Don't know |  |
| 1121. Did the baby have difficulty breathing? | 1.Yes  2.No  98.Don't know | 1126  1126 |
| 1122. How many days after birth did the baby start having  difficulty in breathing? | Days……………………………  98.Don't know |  |
| 1123. Did the baby have chest in drawing? | 1.Yes  2.No |  |
| 1124. Did the baby have grunting? | 1.Yes  2.No  98.Don't know |  |
| 1125. Did the baby have flaring of the nostrils? | 1.Yes  2.No  98.Don't know |  |
| 1126. Did the baby have diarrhea? | 1.Yes  2.No  98.Don't know | 1130  1130 |
| 1127. How many days after birth did the baby have diarrhoea? | Days……………………………  98.Don't know |  |
| 1128. When the diarrhoea was most severe, how many times  did the baby pass stools in a day? | Number…………………………  98.Don’t know |  |
| 1129. Was there blood in the stools? | 1.Yes  2.No  98.Don't know |  |
| 1130. Did the baby have vomiting? | 1.Yes  2.No  98.Don't know | 1230  1230 |
| 1131. How many days after birth did vomiting start? | Days……………………………  98.Don't know |  |
| 1132. When the vomiting was most severe, how many times  did the baby vomit in a day? | Number of times……………………  98.Don't know |  |
| 1133. Did the baby have abdominal distension? | 1.Yes  2.No  98.Don't know |  |
| 1134. How many days after birth did the baby have abdominal  distension? | Days……………………………  98.Don't know |  |
| 1135. Did the baby have redness around, or drainage from, the umbilical cord stump | 1.Yes  2.No  98.Don't know |  |
| 1136. Dd the baby have postural skin rash/ areas of skin that were red, and hot or peeling? | 1.Yes  2.No  98.Don't know |  |
| 1137.Did the baby have yellow palms or soles? | 1.Yes  2.No  98.Don't know |  |
| 1138. How many days after birth did the yellow palms or soles begin? | Days……………………………  98.Don't know |  |
| 1139. For how many days did the baby have yellow palms or soles? | Days……………………………  98.Don't know |  |

**SECTION 12 TREATMENT AND HEALTH SERVICE USE FOR THE FINAL ILLNESS**

| 1201. Did the baby receive any treatment for the illness that led to death? | YES .................................................1  NO…………………………………… 2 | Stop the interview | |
| --- | --- | --- | --- |
| 1202. Can you please list the treatments the baby was given for the illness that led to death?  COPY FROM PRESCRIPTION/DISCHARGE NOTES  IF AVAILABLE |  | |  |
| 1203. Please tell me at which of the following places or facilities  the baby received treatment during the illness that led to death: | Yes No DK  1 Home? 1 2 98  2 Traditional healer? 1 2 98  3 Government clinic? 1 2 98  4 Government hospital? 1 2 98  5 Private clinic? 1 2 98  6 Private hospital? 1 2 98  7 Pharmacy, drug seller,?, 1 2 98  8 Any other place or facility? 1 2 98 | |  |
| 1204.I n the month before death, how many contacts with formal  health services did the baby have? | Numbers …………………………………..  of contacts | |  |
| 1205. Did a health care worker tell you the cause of death? | Yes …………………….............................,……............1  No………………………………………………………….2  Don't know …………………………….........................98 | |  |
| 1206. What did the health care worker say? | ------------------------------------------------------------------------------------------------------------------------------------------------------------------------------------------------------------------------------------------------ | |  |
